# Microbial Spectrum of Acute Encephalitis Syndrome With Special Reference to Non-Japanese Encephalitis Cases

**DOI:** 10.7759/cureus.86266

**Published:** 2025-06-18

**Authors:** Kumari Punam, Bipanchi Mahanta, Achinta K Borthakur

**Affiliations:** 1 Microbiology, Gauhati Medical College and Hospital, Guwahati, IND; 2 Medical Microbiology, Nagaon Medical College, Nagaon, IND; 3 Microbiology, Assam Medical College, Dibrugarh, IND

**Keywords:** acute encephalitis syndrome, dengue virus, leptospirosis, scrub typhus, streptococcus pneumoniae

## Abstract

Background: Northeast India is endemic for Japanese encephalitis (JE), which causes acute encephalitis syndrome (AES). Though there is no specific therapy for JE, many etiological agents of AES are treatable. Hence, this study aims to evaluate the AES cases for their etiologies, laboratory parameters, and associated clinical manifestations.

Methods: A hospital-based prospective observational study was carried out, enrolling all consecutive cases of AES satisfying the World Health Organization (WHO) definition. Blood was tested for the malaria parasite. Cerebrospinal fluid (CSF) samples were processed by Gram staining, India ink staining for *Cryptococcus*, Ziehl-Neelsen staining for acid-fast bacilli (AFB), and culture for other bacteria and fungi*. *Serum and CSF samples lacking the Japanese encephalitis virus (JEV) IgM were processed for the detection of other etiologies. The CSF polymerase chain reaction (PCR) method was used to detect *Enterovirus*, *Haemophilus influenzae*, *Neisseria meningitidis*, and herpes simplex virus, and serum IgM enzyme-linked immunoassay (ELISA) detected scrub typhus, *Leptospira*, dengue, chikungunya, and West Nile antibodies.

Results: Out of 395 AES cases tested, 77 were found to be positive for non-JE etiologies. Of these, dengue virus (n=32/371; 8.6%) was the most common, followed by scrub typhus (n=16/348; 4.6%) and *Leptospira *(n=10/362; 2.8%). Positive cases showed CSF pleocytosis (>5 WBC/cumm) and significantly higher protein level (n=6/11; 55%) and sugar level (n=8/11; 73%) in *Streptococcus pneumoniae. *All cases presented with fever (n=11/11; 100%), followed by altered mental status (n=18/19; 94.7%) and seizure (n=22/32; 68.7%).

Conclusion: The number of non-JE causes of AES in Assam is higher. Scrub typhus, dengue virus, and *Leptospira* are other major infectious etiologies of AES that are treatable. Timely diagnosis of such cases will help reduce AES-related complications and mortality.

## Introduction

Acute encephalitis syndrome (AES) poses a significant public health challenge in India. AES is defined by the World Health Organization (WHO) as the acute onset of fever and a change in mental status, including confusion, disorientation, coma, or inability to talk, and/or new onset of seizures, in any person of any age at any time of year. Simple febrile seizures are excluded [1].

About 68,000 clinical cases of Japanese encephalitis (JE) occur, with a case fatality rate of up to 30% among patients with encephalitis [2]. In India, JE is endemic, and the annual incidence of this condition is over 2,500 cases, resulting in more than 500 deaths. Only 14-18% of reported AES cases are linked to Japanese encephalitis virus (JEV) infection, and the cause of most cases in India is still unidentified [2]. Reports from the northeastern part of India documented 30-45.7% JE positivity among suspected AES cases [3-5]. JE has been endemic in Assam since 1976, and every year, an outbreak occurs in various places of the state [6]. Dibrugarh district is the worst-affected. In a recent trend analysis, 304 JE cases were reported from Dibrugarh district, with incidences ranging between 2.7/100,000 and 5.9/100,000 in the population [6].

The causative agent of AES differs depending on the season and geographical location, with the most common viral pathogens being JEV, followed by dengue virus, West Nile virus, Chandipura virus, measles virus, varicella zoster virus, herpes simplex virus (HSV), and *Enterovirus*. Previous research conducted in Tamil Nadu found that JEV was confirmed as the cause of encephalitis in 27.3% of hospitalized children [7]. However, studies from India have indicated that the main causative agent was not JEV but rather HSV, Epstein-Barr virus (EBV), enterovirus 71, scrub typhus, and co-infections, illustrating the evolving landscape of AES in India [8-11]. Most of the bacterial causes of AES include *Neisseria meningitidis*, *Haemophilus influenzae* type b, *Streptococcus pneumoniae*, *Staphylococcus aureus*, *Klebsiella pneumoniae*, and *Pseudomonas aeruginosa* [12]. If diagnosed early, effective treatment can be initiated. Similarly, other etiological agents like malaria due to *Plasmodium falciparum*, *Rickettsia* causing scrub typhus (*Orientia tsutsugamushi*), and *Leptospira* among the spirochetes have been implicated, which can be treated [13].

Therefore, though there is no specific treatment for JE, many etiological agents of AES are treatable. Hence, the establishment of a non-JE etiology of AES cases is becoming a priority. The present study aims to detect the etiological agents among non-JE AES cases, their clinical manifestations, and their laboratory parameters.

## Materials and methods

A hospital-based prospective observational cross-sectional study design was adopted. A one-year study from June 2017 to July 2018 was conducted in the Department of Microbiology at Assam Medical College, Dibrugarh, Assam, India. All consecutive non-repetitive clinically suspected AES cases of pediatric and adult age groups attending the institution were included. Out of 660 clinically diagnosed AES cases reported in the study period, 395 (205 pediatric and 190 adult cases) were negative for JE and included in the study.

Inclusion and exclusion criteria

Included in the study were clinically suspected AES cases of pediatric and adult age groups attending the institution and giving consent for the study. In contrast, trauma, cerebrovascular accident, metabolic encephalopathy, and known malignancy cases were excluded from the study.

Ethical permission

Ethical permission was obtained from the Institutional Ethics Committee (H) of Assam Medical College (approval number: AMC/EC/PG/11994; date: 06/09/2018).

Sample collection and processing

Demographic and clinical-epidemiological profiles of patients were recorded in a pre-designed proforma.

A plain vial was used to aseptically collect approximately 5 mL of venous blood from the patients. The blood sample was then centrifuged to separate the serum, which was then divided into sterile tubes for additional testing. Blood samples were specifically obtained from patients for whom a lumbar puncture was not feasible or was contraindicated. All samples were gathered under rigorous aseptic conditions. Three hundred and eighty-three serum samples were available for testing. However, only 371 samples could be tested for dengue virus, 341 samples for chikungunya virus (IgM antibody capture enzyme-linked immunoassay (ELISA), Nipah virus (NIV)), 110 samples for NS1 Ag (Panbio Dengue Early ELISA kit, Abbott Point of Care, Abbott Park, Illinois, United States), 339 samples for West Nile virus (InBios West Nile Detect™ IgM Capture ELISA kit, Seattle, Washington, United States), while 395 samples were tested for scrub typhus using the InBios Scrub Typhus Detect™ IgM Capture ELISA kit (Seattle, Washington, United States). Three hundred and sixty-two serum samples were tested for *Leptospira* (Panbio Leptospira IgM ELISA kit, Seattle, Washington, United States). Besides, 395 whole blood samples were tested for the detection of *Plasmodium falciparum* and *Plasmodium vivax* using the SD BIOLINE Malaria Ag P.f/P.v test kit (Abbott Point of Care, Abbott Park, Illinois, United States).

A sample of cerebrospinal fluid (CSF) (approximately 2 mL) was obtained in a sterile leakproof container from clinically diagnosed AES patients by performing a lumbar puncture. The sample was transported to the laboratory immediately. One tube containing 1 mL of CSF was processed for physical, chemical, cytological, and India ink preparation studies. A second tube containing 1 mL of CSF was aliquoted into four tubes and subjected to staining, culture, and molecular testing. If not processed immediately, the sample was kept at -80°C. Out of 276 available CSF samples, 166 were tested by TaqMan real-time polymerase chain reaction (PCR) (pre-standardized PCR kit for respective viruses and bacteria provided by the National Institute of Mental Health and Neuro Sciences (NIMHANS), Bangalore, India). The manufacturer's instructions were followed to identify *Neisseria meningitidis*, *Haemophilus influenzae* type b, and *Streptococcus pneumoniae*. A total of 184 samples for HSV and 50 samples for *Enterovirus* could be tested. All the CSF samples were subjected to Gram staining, Ziehl-Neelsen (ZN) staining, India ink mounting, and conventional bacterial and fungal culture to detect bacterial and fungal pathogens. Biochemical tests were performed on the CSF to check for CSF glucose, protein, and leukocytes. Blood culture was done for all the corresponding CSF samples to rule out sepsis using conventional methods.

Statistical analysis was conducted using the Epi Info 7 software (Centers for Disease Control and Prevention, Atlanta, Georgia, United States). The results were presented in tables, showing percentages and proportions. P-values were calculated using the chi-squared test and Fisher's exact test to assess the significance of the data, with p≤0.05 considered statistically significant.

## Results

Out of 395 AES cases, 383 serum samples and 276 CSF samples were available for the study. Viral etiologies mostly established the dengue virus. Table 1 describes the percentage distribution of viral etiologies of non-JE AES.

**Table 1:** Distribution of viral etiologies of non-JE AES cases ELISA: enzyme-linked immunoassay; CSF: cerebrospinal fluid; PCR: polymerase chain reaction; JE: Japanese encephalitis; AES: acute encephalitis syndrome

Viral etiology	Type of sample	Type of test	Cases tested (n=395)	Positive cases
Dengue virus	Serum	IgM ELISA	371/395 (93.9%)	n=32/371 (8.6%)
NS1 Ag	Serum	ELISA	110/395 (27.8%)	n=1/110 (0.9%)
Chikungunya virus	Serum	IgM ELISA	341/395 (86.3%)	n=0/341 (0%)
West Nile virus	Serum	IgM ELISA	339/395 (85.8%)	n=0/339 (0%)
Herpes simplex virus	CSF	Real-time PCR	184/395 (46.5%)	n=0/184 (0%)
Enterovirus	CSF	Real-time PCR	50/395 (12.6%)	n=0/50 (0%)

Bacterial pathogens determined among serum and CSF samples were mostly scrub typhus (4.6%; n=16/348), followed by *Leptospira *(2.8%; n=10/362) and *Streptococcus pneumoniae *(6.6%; n=11/166). Table 2 describes the percentage distribution of bacterial etiologies of non-JE AES.

**Table 2:** Distribution of bacterial etiologies of non-JE AES cases ELISA: enzyme-linked immunoassay; CSF: cerebrospinal fluid; PCR: polymerase chain reaction; JE: Japanese encephalitis; AES: acute encephalitis syndrome

Bacterial etiology	Type of sample	Type of test	Cases tested (n=395)	Positive (%)
Scrub typhus	Serum	IgM ELISA	348/395 (88.1%)	16/348 (4.6%)
Leptospira	Serum	IgM ELISA	362/395 (91.6%)	10/362 (2.8%)
Streptococcus pneumoniae	CSF	Real-time PCR	166/395 (42%)	11/166 (6.6%)
Neisseria meningitidis	CSF	Real-time PCR	166/395 (42%)	1/166 (0.6%)
Haemophilus influenzae	CSF	Real-time PCR	166/395 (42%)	0/166 (0%)
Pseudomonas aeruginosa	CSF	Bacterial culture	276/395 (69.8%)	2/276 (0.7%)
Acinetobacter lwoffii	CSF	Bacterial culture	276/395 (69.8%)	1/276 (0.3%)
Citrobacter koseri	CSF	Bacterial culture	276/395 (69.8%)	1/276 (0.3%)
Staphylococcus aureus	CSF	Bacterial culture	276/395 (69.8%)	1/276 (0.3%)
Mycobacterium tuberculosis	CSF	ZN staining	276/395 (69.8%)	0/276 (0%)

Among parasites, only one case (0.3%) was positive for *Plasmodium falciparum. *All CSF samples (n=276) tested for fungi were negative. Blood cultures in these patients (276 cases corresponding to the CSF samples) were also negative for bacteria or fungi.

In this study, the distribution of positive cases is significantly higher in the adult population (n=46/77; 59.7%) in comparison to the pediatric population (n=31/77; 40.2%), showing a ratio of 1:1.5 (p=0.023). Distribution of dengue virus and *Streptococcus pneumoniae *was higher in the pediatric population, whereas scrub typhus was more commonly seen among the adults. However, leptospirosis distribution was equal in both age groups. The in-hospital mortality rate was higher in the adult age group (n=12/13; 92.4%) compared to the pediatric group (n=1/13; 7.6%) (p=0.6). However, of the total 77 patients, 39 were not traceable to assess the outcome.

**Figure 1:**
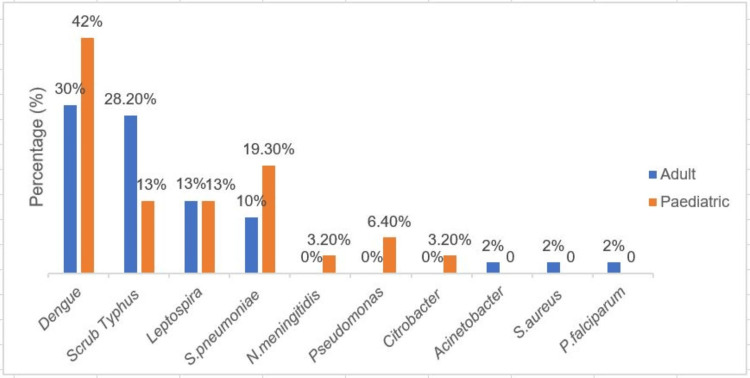
Percentage distribution of etiological agents of non-JE AES as per age group JE: Japanese encephalitis; AES: acute encephalitis syndrome

Figure 1 shows the percentage distribution of various etiological agents of non-JE AES according to age group.

Out of 77 positive cases, 47 cases were male and 30 were female (M:F ratio: 1.5:1), signifying male preponderance. The seasonal prevalence of vector-borne cases showed that the majority of the cases occurred during the monsoon season (July and August). July has a maximum number of cases, 22, with the lowest in the month of December, with one case. 

Biochemical analysis of CSF of positive cases showed increased evidence of CSF pleocytosis (>5 WBC/cumm) and reduced protein level (normal range of CSF protein considered 15-50 mg/dl) among all the positive cases. However, in *Streptococcus pneumoniae* cases, the protein level was significantly elevated (55%). Sugar level (normal range of CSF sugar considered 40-70 mg/dl) was found to be significantly elevated among all the positive cases. Table 3, attached below, shows the CSF parameters among the most common causative agents of AES.

**Table 3:** Laboratory analysis of CSF parameters in the most common causes of non-JE AES CSF: cerebrospinal fluid; JE: Japanese encephalitis; AES: acute encephalitis syndrome

CSF analysis		Dengue-positive cases (n= 22) (%)	Scrub typhus-positive cases (n=15) (%)	*Leptospira*-positive cases (n=8) (%)	*Streptococcus pneumoniae-*positive cases (n=11) (%)
Cell count (cumm)	≤5	10/22 (45%)	3/15 (20%)	3/8 (37.5%)	5/11 (45.4%)
	> 5	12/22 (54%)	12/15 (80%)	5/8 (62.5%)	6/11 (54.5%)
Protein (mg/dl)	≤50	13/22 (59%)	9/15 (60%)	5/8 (62.5%)	5/11 (45%)
	>50	9/22 (41%)	6/15 (40%)	3/8 (37.5%)	6/11 (55%)
Sugar (mg/dl)	≤40	8/22 (36%)	4/15 (26.6)	1/8 (12.5%)	3/11 (27%)
	>40	14/22 (64%)	11/15 (73.3)	7/8 (87.5%)	8/11 (73%)

All cases presented with fever (100%), followed predominantly by a change in mental status (94.7% in bacterial cases and 84.3% in viral cases) and seizure (68.7%). Other features, such as headache (63.1%), unconsciousness (36.3%), and neck rigidity (36.3%), were more commonly associated with bacterial etiologies of AES. Table 4 shows the percentage distribution of different types of clinical features among the most common causes of non-JE AES.

**Table 4:** Clinical presentations in the most common causes of non-JE AES JE: Japanese encephalitis; AES: acute encephalitis syndrome

Clinical features	Dengue (%)	Scrub typhus (%)	*Leptospira *(%)	*Streptococcus pneumonia*e (%)
Fever	32/32 (100%)	19/19 (100%)	12/12 (100%)	11/11 (100%)
Headache	19/32 (59.3%)	12/19 (63.1%)	3/12 (25%)	6/11 (54.5%)
Change in mental status	27/32 (84.3%)	18/19 (94.7%)	9/12 (75%)	4/11 (36.3%)
Seizure	22/32 (68.7%)	8/19 (42.1%)	6/12 (50%)	7/11 (63.6%)
Neck rigidity	7/32 (21.8%)	8/19 (42.1%)	5/12 (41.6%)	5/11 (45.4%)
Paralysis	2/32 (6.25%)	11/19 (57.8%)	1/12 (8.3%)	0/11 (0%)
Unconsciousness	5/32 (15.6%)	4/19 (21%)	1/12 (8.3%)	411 (36.3%)

## Discussion

The clinical features and presentations of various pathogens can lead to similar and overlapping manifestations in AES. This study showed that 77/395 (19.5%) positive cases had non-JE AES as the most common cause, which is in concordance with a study conducted by Desai in NIMHANS, Bangalore, confirming 19% of the samples tested as non-JE pathogens [14].

Out of the five targeted viruses, this study could detect 8.6% (n=32/371) of dengue virus IgM. A similar study from NIMHANS by Ravi et al. showed around 5% cases of dengue virus as the causative agent of AES in Assam [15]. Results for other viruses, namely, chikungunya virus, West Nile virus, HSV, and *Enterovirus* cases, were similar to studies from Assam. Of course, in a study carried out in the northeastern states, 11.8% of cases were positive for the chikungunya virus [16]. Another study conducted by Dutta et al. in Assam did not detect IgM antibodies to the chikungunya virus; however, following real-time PCR, chikungunya virus RNA was detected in 6% of cases [17]. The presence of IgM antibodies to West Nile virus was found in 11.6% of serum samples from patients with non-JE AES in JEV-endemic regions of Assam, as reported previously [18]. In India, enterovirus 71 infection is mainly linked to sporadic disease. However, there is no evidence of enterovirus 71 as a direct causative agent for AES from studies carried out elsewhere [19,20]. Variations in the findings of different authors might be due to the time, location, and outbreak situations in the area.

In this study, among bacterial pathogens causing AES, *Streptococcus pneumoniae* (n=11/166; 6.6%) was most common, followed by scrub typhus (n=16/348; 4.5%) and *Leptospira *(n=10/362; 2.7%). A similar study conducted in NIMHANS from Assam by Ravi et al. shows results concordant with this study, where *Streptococcus pneumoniae* isolated from CSF is 3.5% in AES cases [15]. However, all the cases presented with overlapping symptoms of meningitis, rather than pure encephalitis. Scrub typhus contributed to 4.5% (n=16/348) of the etiologies in this study, which is less in comparison to other studies conducted by Khan et al. in Northeast India and Mittal et al. from North India [21,22]. A study from Tamil Nadu showed a higher prevalence of scrub typhus, followed by dengue [23]. Leptospirosis is yet another cause contributing to AES revealed in this study, accounting for 2.7% (n=10/362) of cases, which is concordant with the previous studies from Assam [15,24]. Other bacteria, namely, *Pseudomonas aeruginosa *(n=2/276; 0.7%), *Acinetobacter lwoffii* (n=1/276; 0.3%), *Citrobacter koseri* (n=1/276; 0.3%), and *Staphylococcus aureus* (n=1/276; 0.3%), showed very low prevalence. A study conducted in Assam by Bhagawati et al. showed similar isolates in cases of meningitis, which were *Escherichia coli* (11.76%), *Acinetobacter *sp. (7.84%), *Pseudomonas *sp. (5.88%), and *Citrobacter *sp. (4%) in CSF samples [25].

A study from the northeast published that 66.2% showed non-JE etiology with scrub typhus (25.7%), mumps (19.5%), measles (4.2%), parvovirus B19 (3.9%), *Plasmodium *(2.7%), HSV 1 and 2 (2.4%), and EBV and *Streptococcus pneumoniae* (2.1% each) being common [26]. *Mycobacterium tuberculosis *also does not show any contribution in the present study.

This study showed the distribution of significantly high non-JE-positive cases in the adult population in comparison to the pediatric population, with a ratio of 1.5:1 (p=0.023). Perhaps the adult population has some protective antibodies against JE due to vaccination programs and some herd immunity living in the endemic zone. The distribution of pathogens shows dengue virus and *Streptococcus pneumoniae* to be higher in the pediatric population. Scrub typhus was reported more among the adult group, whereas leptospirosis was equal in both age groups. The majority of cases were reported during the monsoon season, with the highest number of cases occurring in July, possibly due to an increased prevalence of breeding mosquitoes that thrive during the rainy season.

The in-hospital mortality rate was higher in the adult age group (n=12/13; 92.4%) compared to the pediatric group (n=1/13; 7.6%); however, of the total 77 patients, 39 were not traceable to assess the outcome. Sharma and colleagues stated that there is a lack of information on the local epidemiology and causes of AES in India [27]. Hence, the overall scenario of the outcome resulting from non-JE AES is not comparable to other studies.

Fever with altered mental sensorium was a consistent finding of non-JE AES's viral causes, calling for a broader perspective of clinical suspicion for AES, as most of the cases were treated mostly as JE, whereas, according to this study, signs of meningeal involvement in an AES case mostly pointed toward the bacterial cause of AES.

Strengths of the study

This study describes the various etiologies of AES that are missed mainly by physicians and are potentially treatable. Therefore, it can serve as an eye-opener for the treating physicians to consider while attending to AES cases.

Limitations of the study

As this is a single-centered tertiary-care hospital-based study, an exact depiction of the case load was not possible. Moreover, as this was only a one-year study, the proportion of AES cases may require further studies.

## Conclusions

Even though JE has long been recognized as the primary reason for AES in India, a growing number of recent studies have indicated causes other than JEV, indicating a shift in the AES scenario in India, particularly in the northeastern region. A systematic method for diagnosing encephalitis is necessary for patient management. Non-JE AES are from multiple etiologies, and the optimal diagnostic approach differs depending on the specific agent involved. Enhanced diagnostic techniques are utilized to confirm the causative agents of AES, with a combination of strong clinical suspicion and laboratory confirmation helping to effectively treat the cases, as these infections have definite lines of treatment, unlike symptomatic treatment provided with JE or underdiagnosed cases of AES. Our study showed a varied range of etiologies of non-JE AES, of which dengue, scrub typhus, *Streptococcus pneumoniae*, and *Leptospira* were common. These kinds of studies will also help in understanding the epidemiology of AES and facilitate research and public health disease surveillance.
